# Generation of E-band metasurface-based vortex beam with reduced divergence angle

**DOI:** 10.1038/s41598-020-65230-7

**Published:** 2020-05-19

**Authors:** Hyeongju Chung, Daeik Kim, Ashwini Sawant, Ingeun Lee, Eunmi Choi, Jongwon Lee

**Affiliations:** 1 School of Electrical and Computer Engineering, Ulsan National Institute of Science and Technology, Ulsan, 44919 Korea; 2 Department of Physics, Ulsan National Institute of Science and Technology, Ulsan, 44919 Korea

**Keywords:** Electrical and electronic engineering, Optical materials and structures

## Abstract

Vortex beams carrying orbital angular momentum (OAM) have attracted considerable attention for the development of high-capacity wireless communication systems due to their infinite sets of orthogonal modes. However, the practical applications of Laguerre-Gaussian type vortex beams are limited due to the fact that the divergence angle increases as the order of the OAM mode increases. In this work, we present metasurfaces that generate vortex beams carrying OAM modes with reduced divergence angles in the E-band frequency range. The metasurfaces were designed using eight different meta-atom phase elements, including a spiral phase distribution for OAM modes *l* = 1 and 2, a phase gradient array to avoid interference with the source beam, and a lens pattern array to reduce the divergence angle. Through simulation and experimental measurement, it was confirmed that the divergence angle of the vortex beam generated by the metasurface with the lens pattern was reduced from 13° to 9° and 14° to 11° for OAM modes *l* = 1 and 2, respectively, in comparison with the metasurface without the lens pattern. Our results provide new design methods for various applications based on OAM multiplexing especially in high frequency E-band range.

## Introduction

Metasurfaces are two-dimensional (2D) structures consisting of an array of subwavelength units called meta-atoms. Owing to their superior abilities to control the local phase and scattering amplitude responses in the deep subwavelength scale, metasurfaces have shown great potential for use in building various functional 2D surfaces, such as flat lenses^1–3^, waveplates^4–6^, and surfaces that can control the spin- or orbital-angular momentum (OAM) of electromagnetic (EM) waves^7–11^, as well as in holography^12–15^, vortex beam generation^7,9–11^, and so on. Among the above-mentioned applications, a vortex beam carrying nonvanishing OAM mode based on a metasurface was first implemented in 2011 by Yu *et al*.^7^, using a phase array of V-shaped subwavelength antennas. A vortex beam exhibits a helical phase response expressed as exp(*ilϕ*), where *ϕ* is the azimuthal angle and *l* is the state number of the OAM mode^16^. A vortex beam can carry multiple orthogonal OAM modes, which can significantly enhance the data capacity when each OAM mode is used as a data channel^17–21^. Consequently, vortex beams are receiving considerable attention, especially in the field of wireless communication.

Generation of vortex beam carrying OAM modes based on a metasurface can be implemented using the abrupt local phase discontinuities occurring at the meta-atom for an incident EM wave. Two methods are commonly used for the phase-front engineering of metasurfaces^1,22^. The first method is to control the phase of the scattered field from the meta-atom by adjusting the structural parameters of the meta-atom under linearly polarized EM wave illumination. In this approach, it is necessary to design several meta-atom structures for each local phase required for phase front engineering. The second method is to use a local phase response change from the in-plane spatially varying orientation of an isotropic subwavelength meta-atom under illumination by a circularly polarized incident EM wave. Using the above-mentioned methods, various metasurface structures for vortex beam generation have been studied in the recent past; however, due to the unavoidable beam divergence of vortex beams with the characteristics of Laguerre-Gaussian (LG) type waves^16,23–26^, their practical implementation in free-space wireless-communication system is limited. Recently, several studies focused on reducing the divergence angles of OAM beams by applying meta-lens structures in the radio frequency (RF) range have been reported^26–29^. To manage the explosive increase in data capacity, research has been conducted on the use of communication links based on OAM multiplexing in the millimeter E-band frequency range^30,31^, but studies on controlling the divergence angles of OAM beams in this frequency range have not been conducted yet.

Here, we propose the generation of vortex beams with reduced divergence angles in the E-band frequency range (80–85 GHz) using a metasurface in which multiple pattern arrays are integrated. The metasurface developed in this work utilizes a spiral phase meta-atom array to produce OAM modes, a one-dimensional (1D) gradient phase array for beamsteering so that it can be used in combination with a source in the E-band range, and a meta-lens array to reduce the divergence angle of the OAM mode carrying the vortex beam. Considering that the data capacity is growing rapidly and demands for the use of higher carrier frequencies are increasing, our metasurface may be applied to construct OAM mode multiplexing-based front-haul or back-haul wireless communication systems.

## Results and Discussion

### Metasurface design

**Figure 1:**
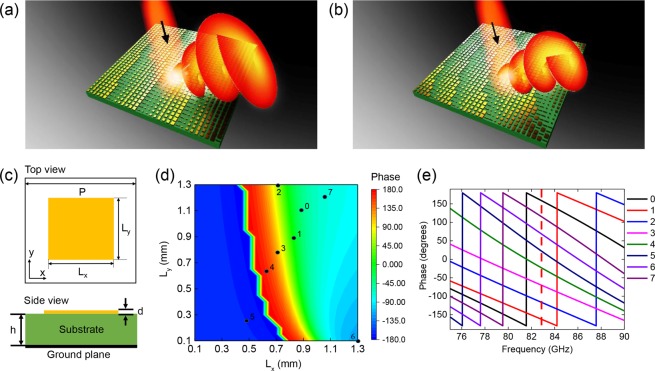
Schematics of vortex beam generation from (**a**) a metasurface with a spiral phase and 1D gradient array and (**b**) a metasurface with a spiral phase array, 1D gradient array, and lens structure. (**c**) Top and side views of meta-atom unit structure. The dimensions of the structure are *d* = 0.018 mm, *h* = 0.25 mm, and *P* = 1.5 mm. (**d**) Simulated reflection phase for different lengths of *L*_*x*_ and *L*_*y*_ of the square patch antenna for *x*-polarized normal incidence beam at 83 GHz. The black dots and numbers indicate the selected eight different meta-atom unit structures. (**e**) Simulated reflection phase for the eight-unit structures as a function of frequency (75–90 GHz). The eight-unit structures were optimized to have 45° reflection phase differences from each other at 83 GHz.

Schematics of OAM mode-carrying vortex beam generation using different metasurfaces are depicted in Fig. 1(a,b). The metasurface shown in Fig. 1(a) was designed by integrating a spiral-phase meta-atom array with a 1D gradient phase array, generating a vortex beam with a specific OAM mode for a linearly polarized Gaussian input beam. The metasurface structure depicted in Fig. 1(b) was designed by including a meta-lens structure in addition to the structure of Fig. 1(a), in which the generated vortex beam has a reduced divergence angle compared to that generated using the previous structure. For the metasurface design, the reflective square patch antenna structure shown in Fig. 1(c) was used as the meta-atom unit structure and the structure was designed to respond to a linearly *x*-polarized normal incidence beam at 83 GHz, which is in the E-band. The top square patch antenna and bottom ground plane were composed of copper, and a dielectric substrate (Taconic RF-35A2) with a loss tangent of 0.0015 was sandwiched between the two metallic layers. The geometric parameters are provided in the figure captions. For the phase front engineering, eight different meta-atom structures with 45° local phase response spacing were designed by adjusting the square patch antenna lengths *L*_*x*_ and *L*_*y*_ through numerical simulations (see the Methods section for the simulation details). Figure 1(d) shows the simulated local reflection phase responses obtained by sweeping *L*_*x*_ and *L*_*y*_ from 0.1 mm to 1.3 mm in 0.05 mm steps for *x*-polarized normal incidence beam. Eight different meta-atom units with 45° phase spacing at the target frequency of 83 GHz were chosen to cover the 2π phase response and are marked in Fig. 1(d). Figure 1(e) plots the phase response of the eight meta-atom units as functions of the frequency in the E-band range. As indicated by the dotted red line, the eight meta-atom units have 45° of phase spacing at 83 GHz and the phase spacing remains fairly constant in the 75–90 GHz frequency range, which means that the proposed structures are capable of broadband operation in that range. The reflection amplitudes of the eight units were also monitored by simulation, and the reflection efficiency was confirmed to be more than 90% for all structures, as shown in Supplementary information Fig. S1. Based on the recently demonstrated digitized coding metasurface design approach^11^, the eight meta-atom unit cells with 45° phase spacing were designated as 0 to 7 using the 3-bit digit codes 000 to 111.

**Figure 2:**
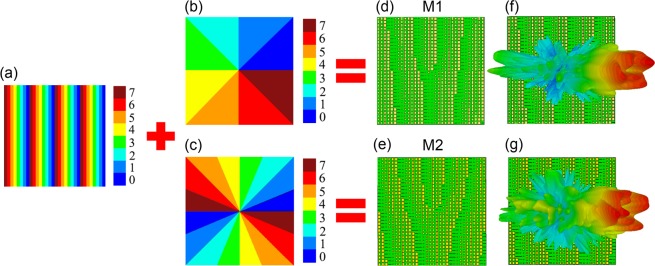
(**a**) 1D gradient phase sequence pattern using the eight different meta-atom unit structures indicated in Fig. 1(d). (**b,c**) Spiral phase patterns for OAM mode (**b**) *l* = 1 and (**c**) *l* = 2 generation. (**d,e**) Vortex beam with OAM mode (**d**) *l* = 1 (M1) and (**e**) *l* = 2 (M2) generation metasurfaces incorporating the 1D gradient and spiral phase patterns. (**f,g**) Far-field scattering patterns of the vortex beams with OAM modes (**f**) *l* = 1 and (**g**) *l* = 2 generated by the metasurface M1 and M2 structures, respectively.

Based on the Fourier transform relationship between the coding pattern and its far-field radiation pattern, the convolution operation used in signal processing could be applied to the metasurface design using the coding patterns^11^. Considering the operating frequency and wavelength of our reflective metasurface, a 1D gradient pattern array with the coding sequence “7, 6, 5, 4, 3, 2, 1, 0, …,” as shown in Fig. 2(a), was used to adjust the reflection beam steering angle to the vortex beam generation metasurface to avoid interference between the vortex beam and the source caused by reflection in the direction normal to the metasurface. Figure 2(b,c) show spiral phase distributions using the 3-bit digit code for the generation of vortex beams carrying OAM modes *l* = 1 and 2, respectively, following the azimuthal phase profile of OAM mode *l* expressed as exp(*ilφ*), where *φ* is the azimuthal angle around the beam axis. The 2D domain is divided into eight sections for *l* = 1 and sixteen sections for *l* = 2, where the 3-bit unit cells are distributed counterclockwise in the order 0, 1, 2, 3, 4, 5, 6, 7. The metasurface is composed of a 32 × 32 array of 3-bit digit codes. Figure 2(d,e) show the designed metasurfaces utilizing a gradient pattern array in the *x*-direction and a spiral phase pattern for generating a vortex beam with OAM modes *l* = 1 (M1) and *l* = 2 (M2), respectively. From the generalized Snell’s law, the reflection angle *θ*_*r*_ of the vortex beam generated by a metasurface with the 1D gradient pattern array can be expressed as

1 $${\theta }_{r}={\sin }^{-1}(\sin \,{\theta }_{i}+\lambda /\varGamma )$$

where a gradient pattern period Γ of 12 mm and an input beam incidence angle *θ*_*i*_ of 15° were used. In this configuration, the reflection angle *θ*_*r*_ from the z-axis was calculated to be 33.97°. The simulated far-field radiation patterns generated by metasurfaces M1 and M2 are shown in Fig. 2(f,g), respectively. The two far-field radiation patterns both exhibit the deep null region at the center of the beam and the reflection angle of the vortex beam of *θ*_*r*_= 33° is obtained for the Gaussian input beam with *θ*_*i*_ = 15°, which is well matched with the theoretical prediction.

**Figure 3:**
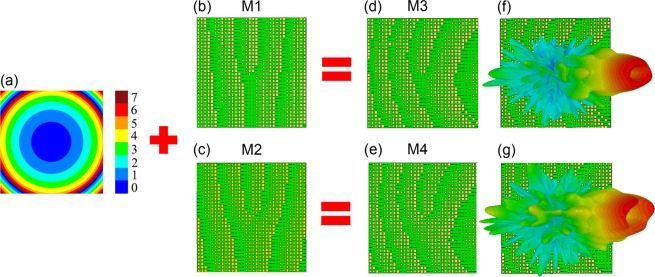
(**a**) Lens structure phase pattern obtained using the eight different meta-atom unit structures. (**b,c**) M1 and M2 metasurface structures. (**d**) Metasurface structure (M3) incorporating the lens structure with the M1 metasurface structure for generation of OAM *l* = 1 mode with reduced divergence angle. (**e**) Metasurface structure (M4) incorporating the lens structure with the M2 metasurface structure for generation of OAM *l* = 2 mode with reduced divergence angle. (**f,g**) Far-field scattering patterns of the vortex beams with OAM modes (f) *l* = 1 and (g) *l* = 2 generated by the metasurface M3 and M4 structures, respectively.

A vortex beam carrying OAM modes is an LG wave, and the divergence angle of the beam increases as the topological charge *l* increases^24^. The divergence angle of the main lobe of an OAM beam can be a major disadvantage in long-distance communication applications. To reduce the divergence angle of the OAM beam, the lens pattern shown in Fig. 3(a) was incorporated into the previously designed metasurface structures M1 and M2 as shown in Fig. 3(b,c), respectively, and the resulting metasurface structures M3 and M4 for OAM modes *l* = 1 and 2 are shown in Fig. 3(d,e), respectively. For the lens structure design, the local phase distributions were obtained from the following lens phase equation:

2 $${\varPhi }_{L}(x,y)=\frac{2\pi }{\lambda }(\sqrt{{F}^{2}+{x}^{2}+{y}^{2}}-{F}^{2})$$

where *λ*= 3.6 mm is the operating wavelength, *x* and *y* are spatial coordinates, and *F* = 131 mm is the focal length of the lens. The focal length was obtained by calculating the focal length required to collimate a Gaussian input beam with an 8 mm beam waist located 100 mm away from the metasurface. We note that to obtain the minimum divergence angle of the OAM beam, meta-lens structure having an optimal focal length for each OAM mode should be used, and in the case of OAM mode *l* = 1 and 2 for this work, the minimum divergence angle of the two OAM beams were achieved when applying the meta-lens structure with focal length of *F* = 131 mm (see Supplementary information Fig. S11,S12). The simulated far-field radiation patterns of the vortex beams generated by metasurfaces M3 and M4 are shown in Fig. 3(f,g), respectively, where the deep null region at the center of the beam and the beam-reflection angle are the same as those extracted from the previous case. The reduced divergence angles of the two vortex beams were compared with those generated from the metasurfaces without the lens pattern through the simulation and measurement results, and the detailed results are described in the next section.

### Experimental demonstration

**Figure 4:**
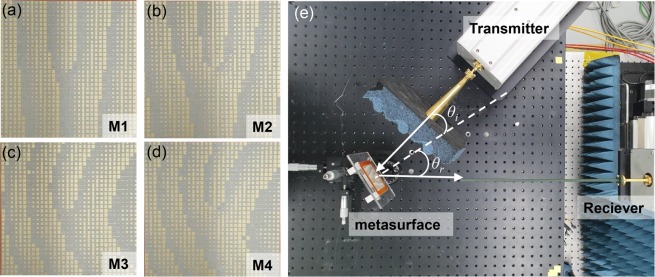
(**a–d**) Photographs of four metasurfaces ((**a**) M1, (**b**) M2, (**c**) M3, and (**d**) M4) fabricated using the PCB process. (**e**) Experimental setup for near-field scattering pattern measurement. The horizontally polarized Gaussian input beam from the transmitter is incident on the metasurface with an incidence angle of *θ*_*i*_ = 15° and the vortex beam generated by the metasurface is reflected at an angle of *θ*_*r*_ = 33° and collected by the receiver with the near-field probe. By horizontal and vertical scanning of the near-field probe, the intensity and phase distribution of the vortex beam were measured.

**Figure 5:**
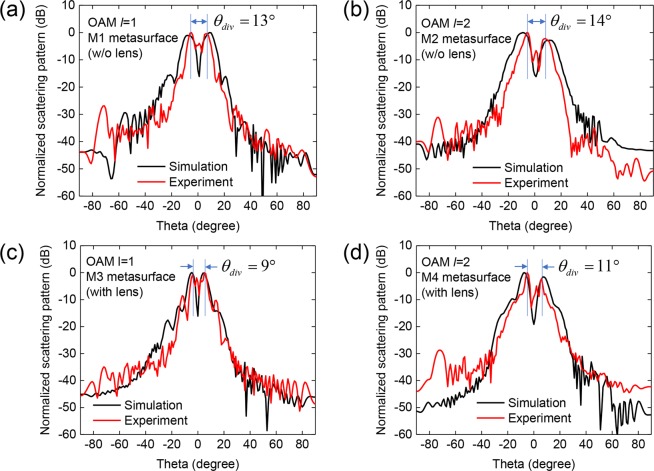
Simulated (black) and measured (red) 2D scattering patterns of the vortex beams at 83 GHz generated by metasurfaces (**a**) M1, (**b**) M2, (**c**) M3, and (**d**) M4.

**Figure 6:**
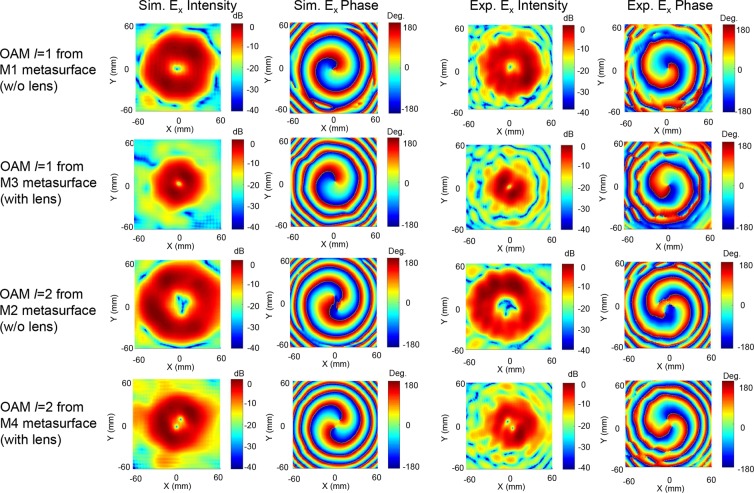
Simulation and measurement results of the intensity and the phase distribution of the E_x_ field of the vortex beam. The first, second, third, and fourth columns indicate simulated *E*_*x*_ field intensity, simulated *E*_*x*_ field, measured *E*_*x*_ field intensity, and measured *E*_*x*_ field phase distribution, respectively. The first, second, third, and fourth rows indicate the vortex beam generated from the M1, M3, M2, and M4 metasurface, respectively.

For experimental demonstration of the proposed concepts and structures, the four designed metasurface structures (M1, M2, M3, and M4) were fabricated via a printed circuit board (PCB) process. Figure 4(a–d) show photographs of the fabricated metasurfaces, each of which consists of a 32 × 32 element array and has actual dimensions of 48×48 mm^2^. Figure 4(e) shows the measurement setup. A linearly polarized Gaussian beam with an 8 mm beam waist is generated by the horn-antenna-equipped transmitter and is incident on the metasurface with an incidence angle of *θ*_*I*_ = 15°. The distance between the metasurface and the transmitter is 100 mm. The vortex beam generated by the metasurface is directed to the receiver equipped with a receiving antenna and near-field probe. The distance between the metasurface and the near-field probe receiving antenna is 150 mm. The transmitter and receiver are connected to two ports of a vector network analyzer (VNA). By scanning the near-field probe in the *x* and *y* directions in 0.6 mm steps, a 120×120 mm^2^ area of the intensity and the phase distribution of the *E*_*x*_ component of the generated vortex beam were measured. Figure 5(a–d) show the simulated and measured 2D far-field scattering patterns of the vortex beams generated by the four metasurfaces at 83 GHz. The simulated and measured 2D scattering patterns in the far-field were transformed from the near-field data using ‘MATLAB near-field to far-field transformation code’. The measured OAM mode vortex beams for the four metasurface structures are in good agreement with the simulated results. Figure 5(a,c) show the 2D far-field scattering pattern of the vortex beams with OAM mode *l* = 1 generated by metasurfaces without and with lens patterns, respectively. The divergence angle *θ*_*div*_ of the vortex beam was measured as the angle between the peak positions of the two main lobes. As can be seen in the two figures, when the lens pattern is applied, the divergence angle *θ*_*div*_ of the OAM beam with *l* = 1 is reduced from 13° to 9°. Similarly, in the generation case with OAM mode *l* = 2 as shown in Fig. 5(b,d), when the lens pattern is applied, the divergence angle *θ*_*div*_ of the OAM beam is reduced from 14° to 11°. In order to characterize the vortex beams generated by the four metasurface structures, the intensity and phase distributions of *E*_*x*_ at 83 GHz were simulated and experimentally measured 150 mm away from the metasurface and the results are shown in Fig. 6. The simulation and experimental measurement results are in good agreement. The spiral phase distributions of *E*_*x*_ obtained from the four metasurface structures clearly indicate that the vortex beams contain OAM modes *l* = 1 and 2. It can be seen that the size of the mode intensity distribution of the OAM beam increases as the OAM mode increases, and the size of the mode intensity can be significantly reduced by using the metasurface with the lens structure. As shown in Fig. 1(e), our metasurface structure can be used in the broadband frequency range from 75 GHz to 89 GHz, and the intensity and phase distribution measurement data at 75, 77, 80, 83, 86, and 89 GHz are provided in Supplementary information Fig. S2–S9. We note that the divergence angle reduction using the meta-lens can be applied to metasurfaces that generate OAM mode higher than *l* = 2. In this case, the minimum divergence angle can be obtained using the meta-lens structure with optimum focal length for each OAM mode. To support this, we show the simulated *E*_*x*_ field intensity and phase distribution of the OAM mode *l* = 3 and 4 from the metasurface in Fig. S10, and the 2D scattering patterns and divergence angles for the first 4 OAM modes from the metasurface with meta-lens structure with seven different focal lengths in Figs S11 and S12, respectively.

## Conclusions

In this work, we proposed and experimentally demonstrated the generation of vortex beams carrying OAM modes *l* = 1 and 2 with reduced divergence angles in the E-band frequency range based on metasurfaces utilizing spiral phase arrays engineered to generate the OAM modes, a 1D gradient array to prevent interference between the vortex beam generated in reflection and the input source antenna, and a lens pattern to reduce the divergence angle of the vortex beam. For comparative analysis, a total of four metasurface structures, including two metasurface structures without lens patterns for OAM *l* = 1 (M1) and *l* = 2 (M2) generation and two-metasurface structures with lens patterns for OAM *l* = 1 (M3) and *l* = 2 (M4) generation, were designed and fabricated. Using the four metasurface structures, the divergence angle of the OAM beam was reduced from 13° to 9° and from 14° to 11° for OAM modes *l* = 1 and 2, respectively, according to the simulation and experimental measurements. The metasurface proposed in this study may provide a new design method of constructing OAM-beam-based long-distance wireless communication systems in the E-band frequency range based on the advantages of the generation of OAM beams with reduced divergence angles.

## Methods

### Numerical simulations

A commercial Maxwell’s equation solver (CST Studio) based on the finite integration method in the frequency domain was used to design the meta-atom unit structures and full metasurface array simulations. In the meta-atom unit structure simulations, the unit cell boundary conditions were used in the *x* and *y* directions, Floquet boundary conditions with Floquet modes TE_00_ and TM_00_ were used in the +*z* direction, and perfect electric conductor boundary conditions were used in the -*z* direction to extract only the reflection coefficient of the antenna pattern. The tetrahedral mesh with 10 divisions for the meta-atom structure and 4 divisions for the background per wavelength was used. By sweeping the geometric parameters, *L*_*x*_ and *L*_*y*_ of the square patch antenna, as well as the amplitude and phase of the reflection coefficient, were extracted. For the full metasurface array simulations, a time domain solver was used, and open boundary conditions were applied in all directions. The linearly *x*-polarized Gaussian beam at 83 GHz with a beam waist of 8 mm and located 100 mm away from the metasurface was used as the input EM source, and the input beam was simulated to be incident onto the metasurface at an incidence angle of 15°. The near-field patterns were extracted using field monitors and the far-field patterns were transformed from the near-field data by using ‘MATLAB near-field to far-field code’.

### Measurement setup

For the 2D scattering pattern measurement, the experimental setup shown in Fig. 4(e), which was composed of a VNA, extender, waveguide twist, feeding source antenna, field probe and motion controller, was used. To minimize possible parasitic reflections, an EM field absorber was placed around the source antenna. In the transmitter part, an extender (OML, Inc., WR10) was connected to the VNA (PNA-X5247A) to generate a vertically polarized beam with an operational frequency range of 75–110 GHz. The waveguide twist, which can convert a vertically polarized beam into a horizontally polarized beam, and the Gaussian horn antenna with a beam waist of 8 mm were connected to the extender. The metasurface was illuminated by the horizontally polarized Gaussian beam from the antenna located 100 mm away from the metasurface, with an angle of 15°. The vortex beam from the metasurface was generated with a reflection angle of 33° and was directed toward the receiving field probe. For the receiver part, an extender (OML, Inc., WR10) with a waveguide field probe was connected to the VNA. The intensity and phase of the vortex beam were extracted by measuring the reflection scattering parameters.

## Supplementary information

Supplementary information.
