# Supplementary material for: Generation of E-band metasurface-based vortex beam with reduced divergence angle
Source: Sci Rep. 2020 May 19;10:8289. doi: 10.1038/s41598-020-65230-7 (PMC7237678; doi:10.1038/s41598-020-65230-7)
Supplement: Supplementary file 1 — Supplementary information. [file 41598_2020_65230_MOESM1_ESM.doc]

**Supplementary Information**

**Generation of E-band metasurface-based vortex beam with reduced divergence angle**

**Hyeongju Chung^1^, Daeik Kim^1^, Ashwini Sawant^2^, Ingeun Lee^2^, Eunmi Choi^1,2^, and Jongwon Lee^1*^**

^1^ School of Electrical and Computer Engineering, Ulsan National Institute of Science and Technology, Ulsan, 44919, Korea

^2^ Department of Physics, Ulsan National Institute of Science and Technology, Ulsan, 44919, Korea

*Corresponding Author: Jongwon Lee (E-mail: [jongwonlee@unist.ac.kr](mailto:jongwonlee@unist.ac.kr) )

**Figure S1**. Simulated reflectance of the meta-atom structure (Figure 1(c) in the main manuscript) for the different square patch antenna lengths *L_x_* and *L_y_* at 83 GHz.


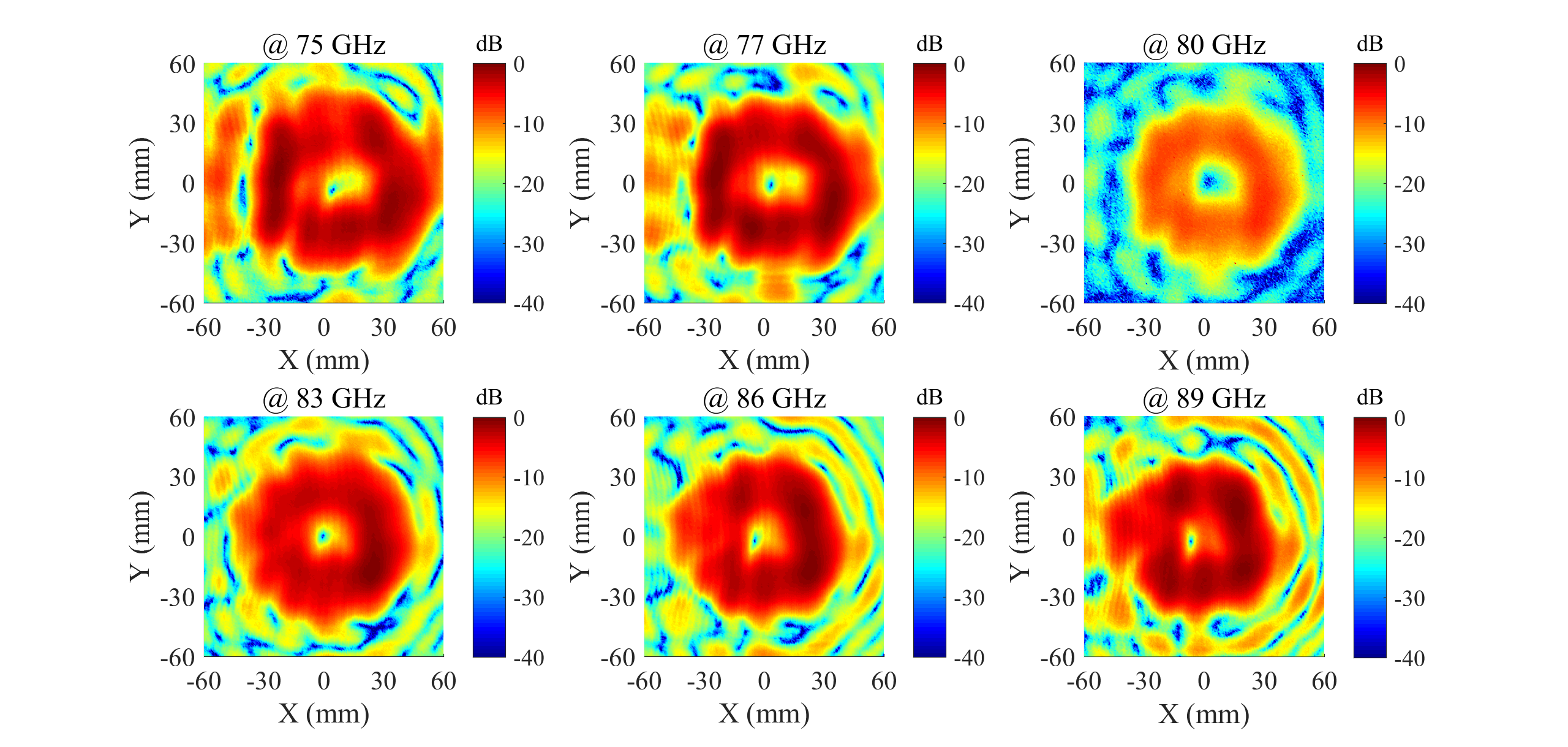


**Figure S2**. Measured *E_x_* field intensity distribution of the vortex beam with OAM mode *l* = 1 generated by the M1 metasurface (without lens structure) at 6 different E-band frequencies.


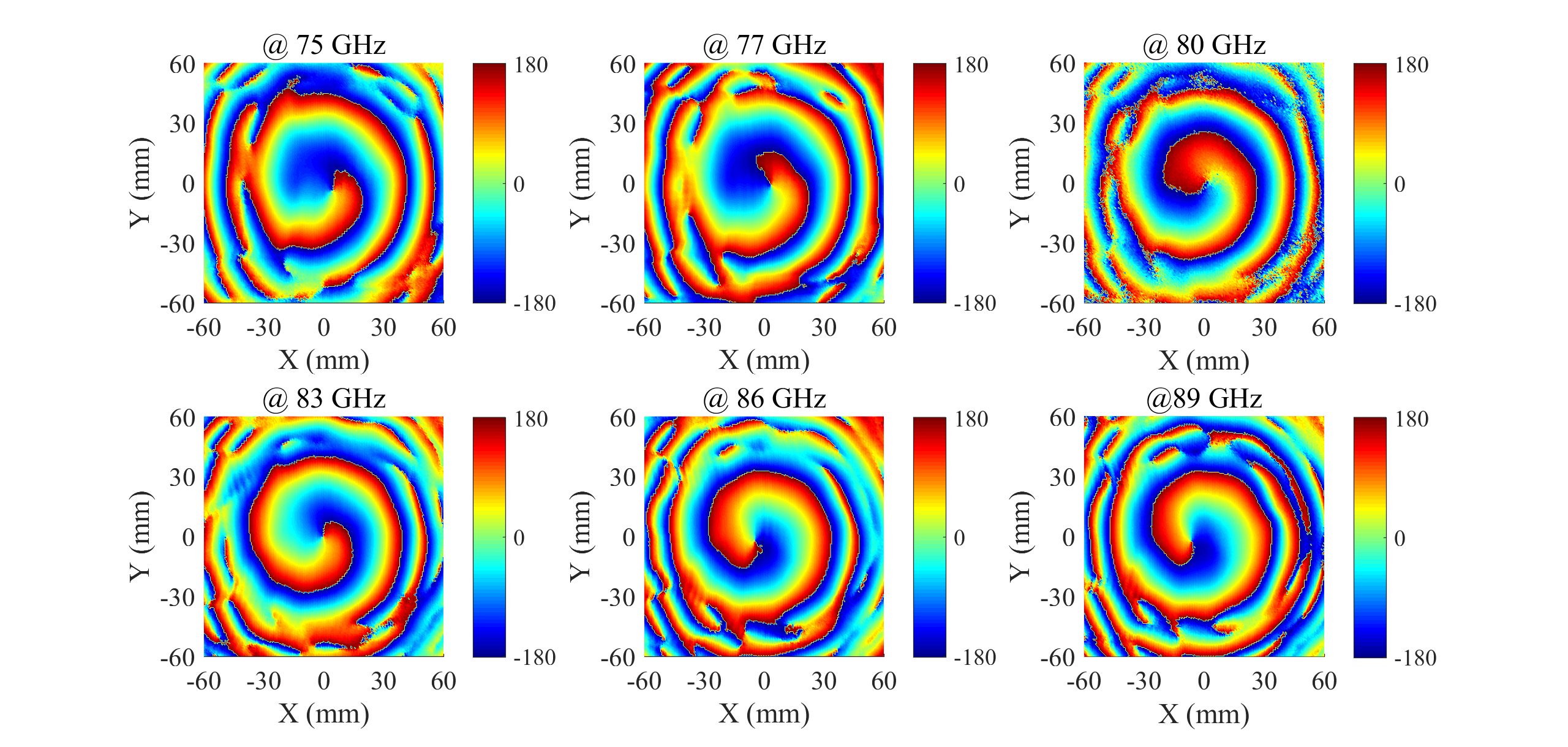


**Figure S3**. Measured *E_x_* field phase distribution of the vortex beam with OAM mode *l* = 1 generated by the M1 metasurface (without lens structure) at 6 different E-band frequencies.


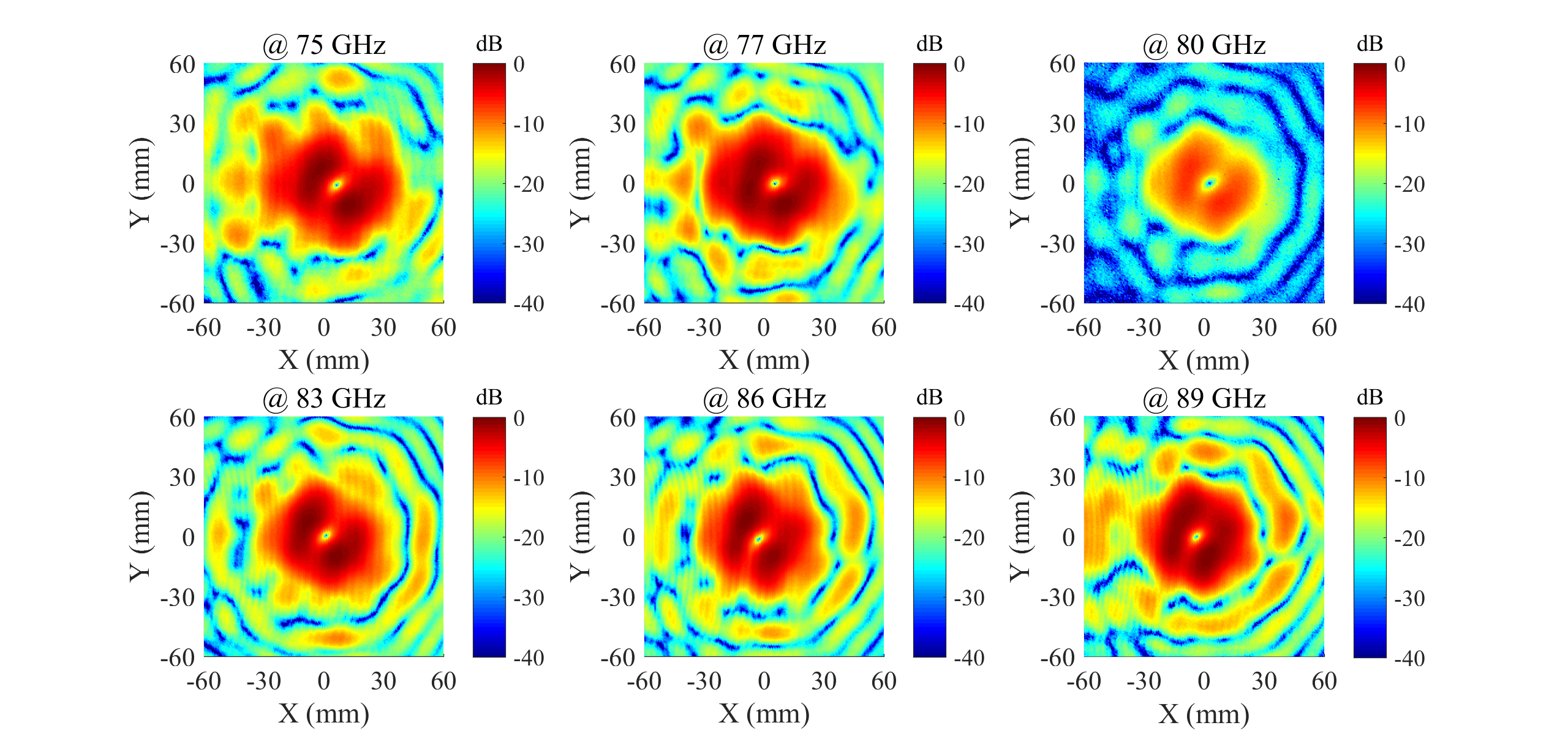


**Figure S4**. Measured *E_x_* field intensity distribution of the vortex beam with OAM mode *l* = 1 generated by the M3 metasurface (with lens structure) at 6 different E-band frequencies.


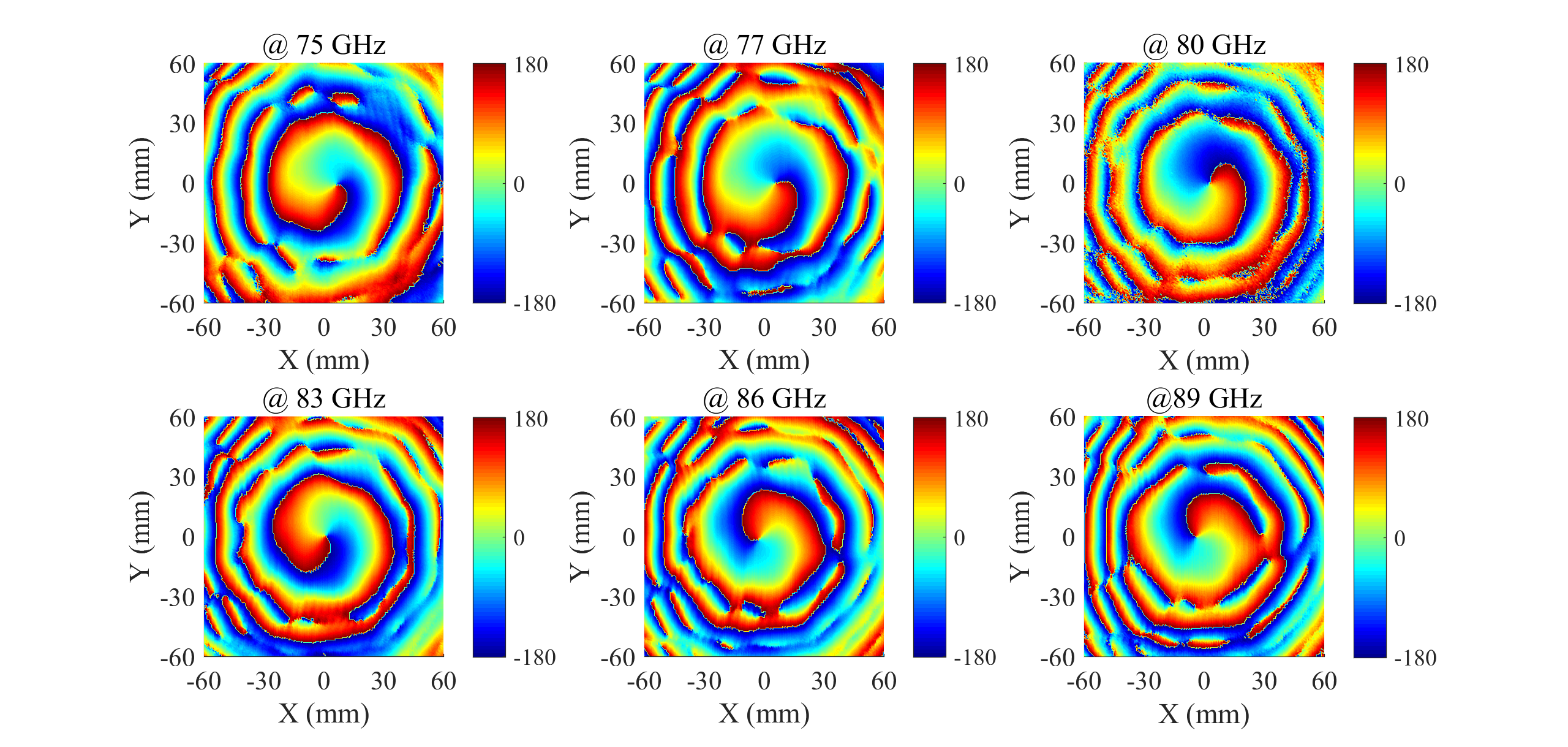


**Figure S5**. Measured *E_x_* field phase distribution of the vortex beam with OAM mode *l* = 1 generated by the M3 metasurface (with lens structure) at 6 different E-band frequencies.


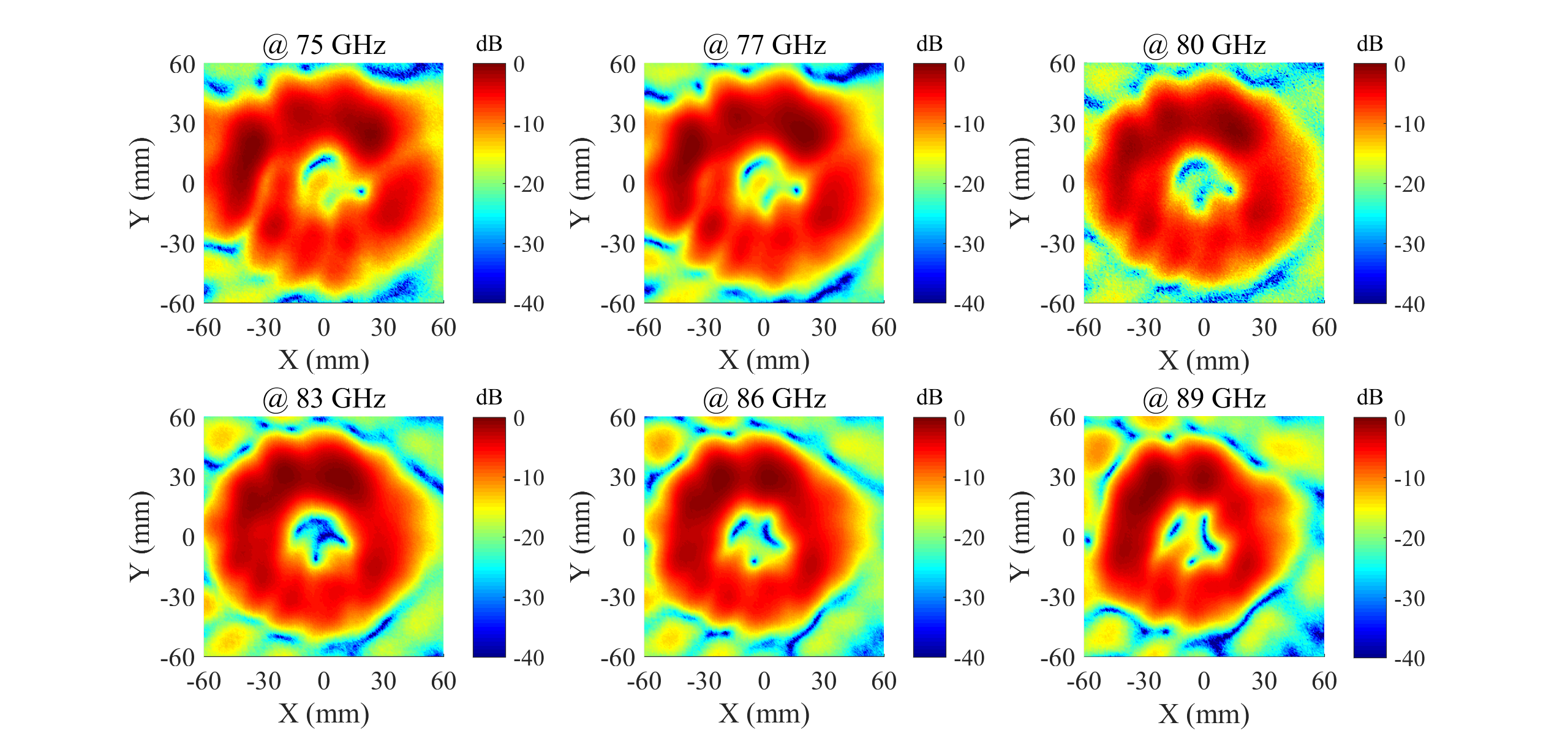


**Figure S6**. Measured *E_x_* field intensity distribution of the vortex beam with OAM mode *l* = 2 generated by the M2 metasurface (without lens structure) at 6 different E-band frequencies.


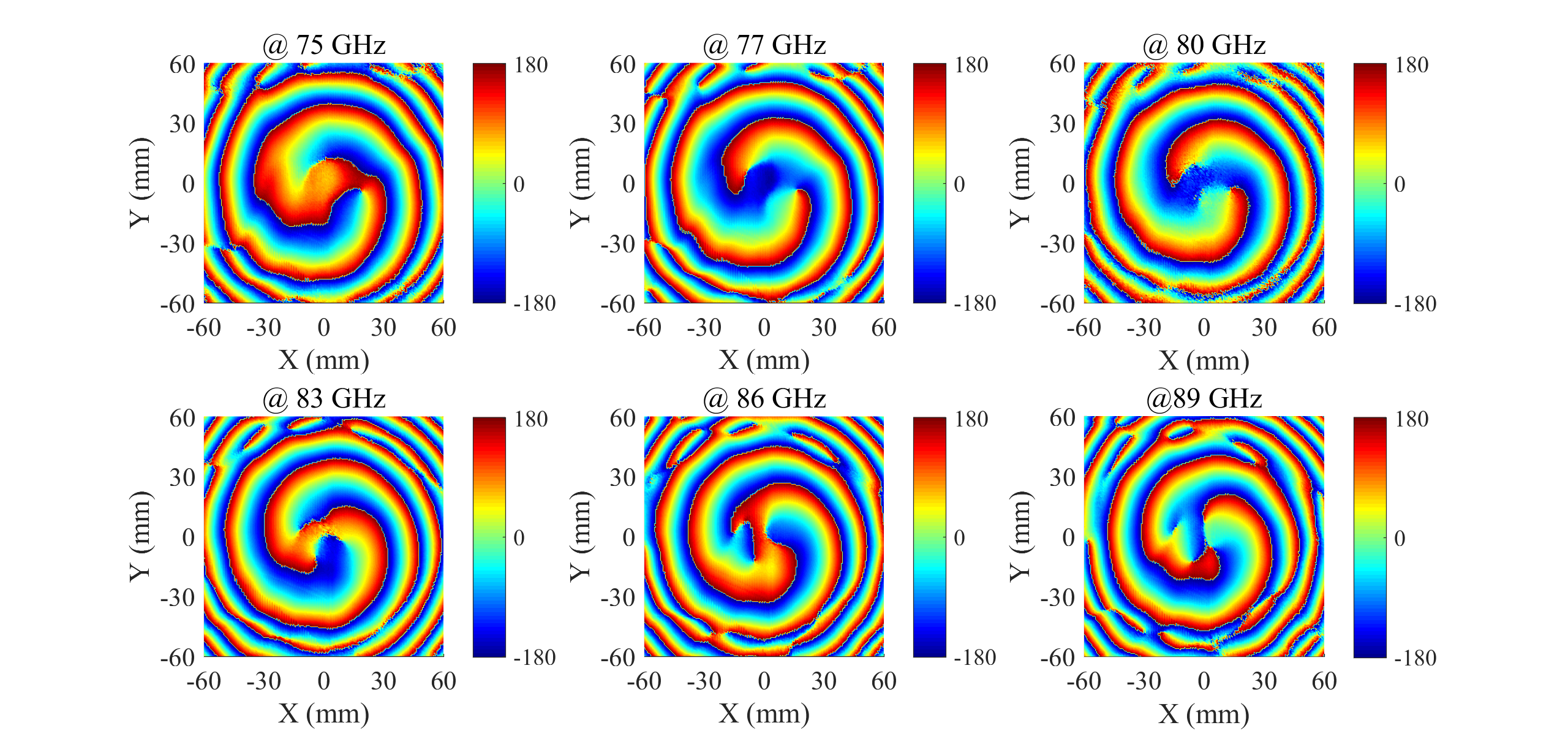


**Figure S7**. Measured *E_x_* field phase distribution of the vortex beam with OAM mode *l* = 2 generated by the M2 metasurface (without lens structure) at 6 different E-band frequencies.


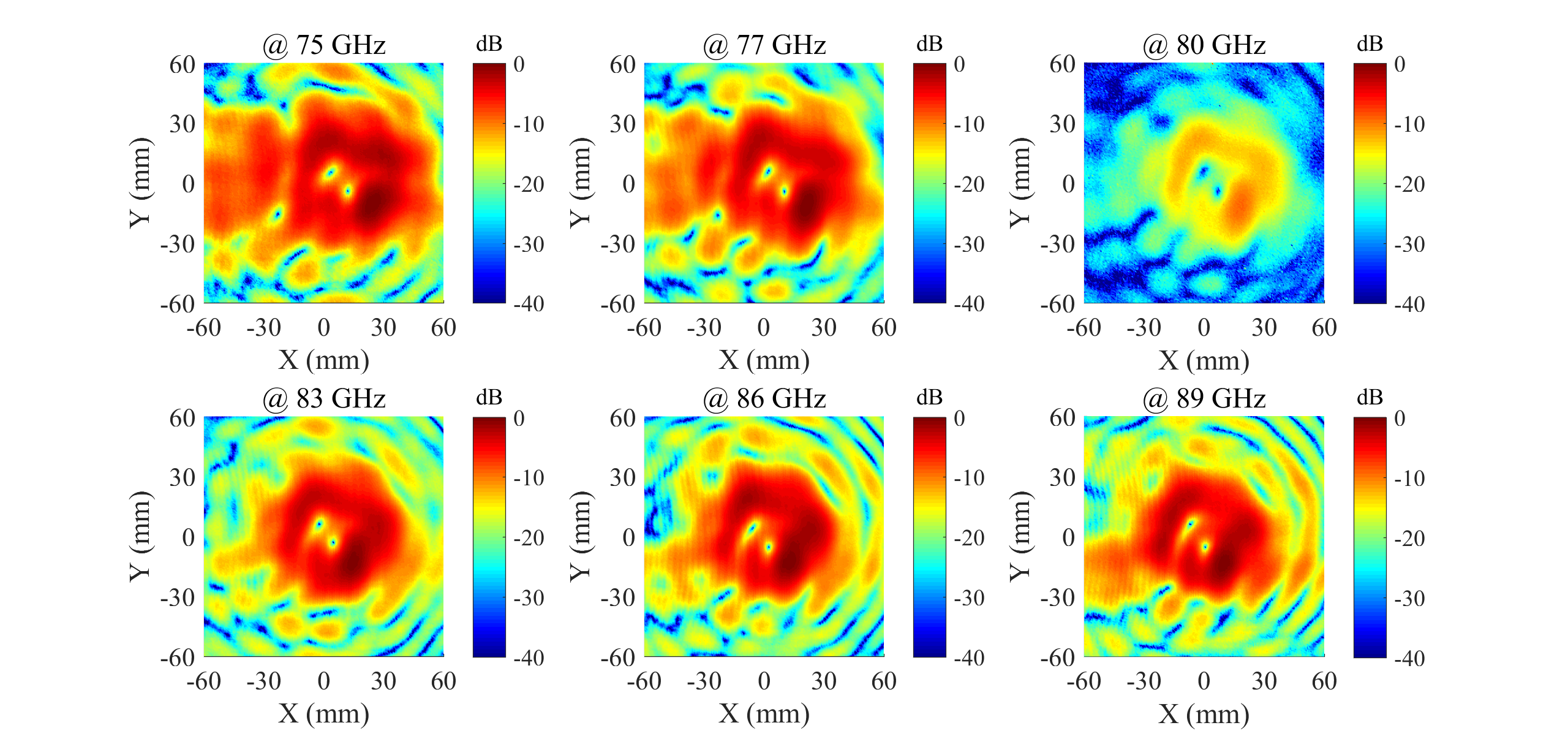


**Figure S8**. Measured *E_x_* field intensity distribution of the vortex beam with OAM mode *l* = 2 generated by the M4 metasurface (with lens structure) at 6 different E-band frequencies.


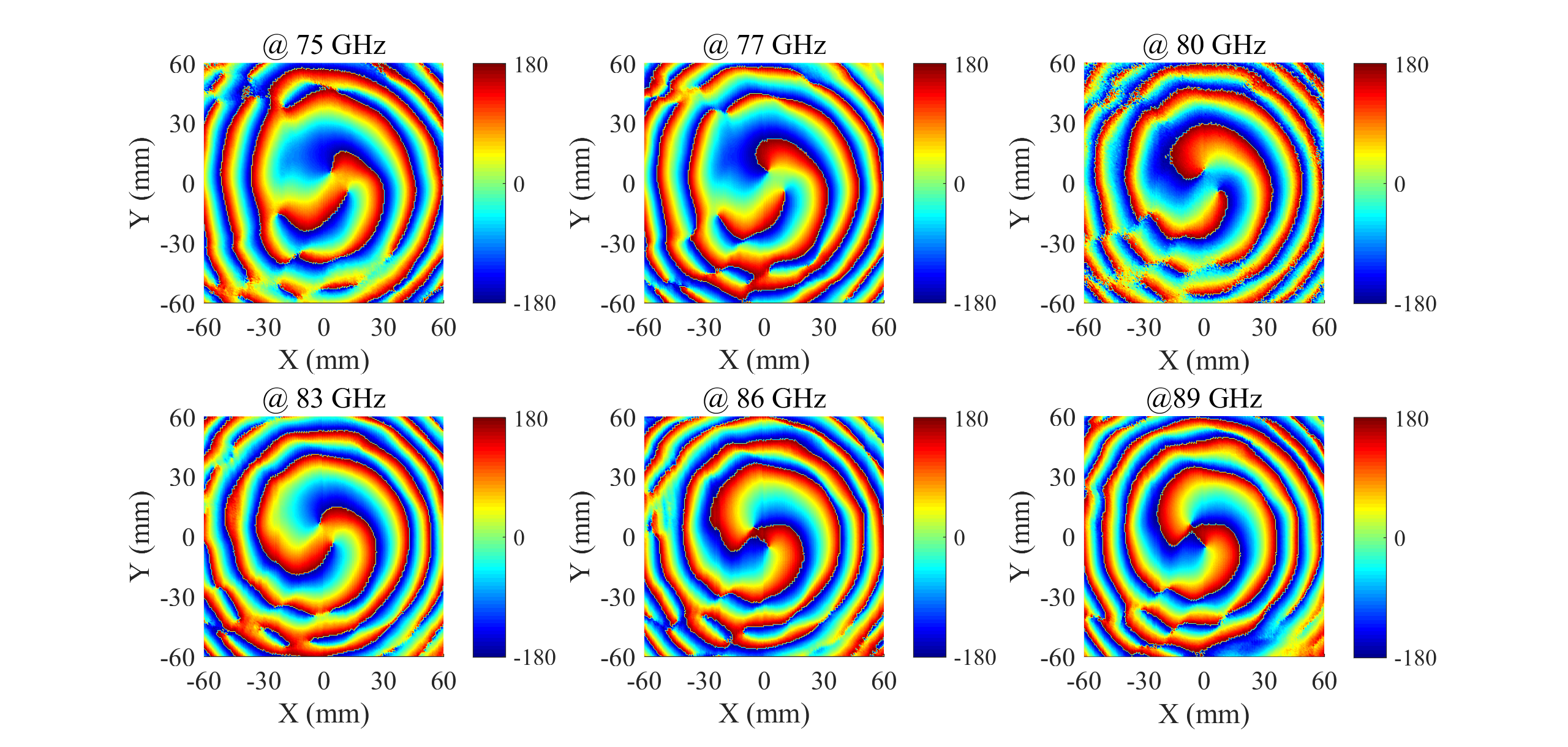


**Figure S9**. Measured *E_x_* field phase distribution of the vortex beam with OAM mode *l* = 2 generated by the M4 metasurface (with lens structure) at 6 different E-band frequencies.


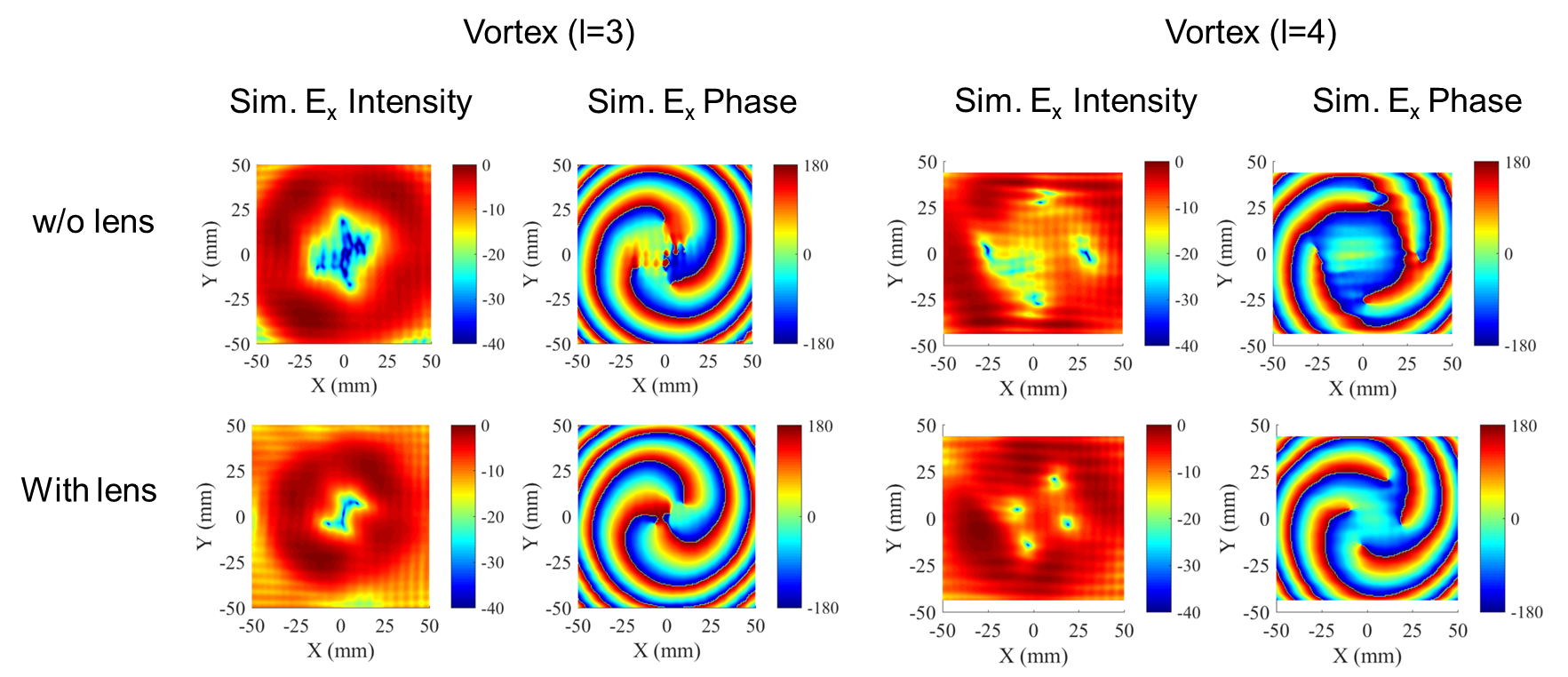


**Figure S10**. Simulation results of the intensity and the phase distribution of the E_x_ field of the vortex beam. The first and second columns indicate simulated E_x_ field intensity and phase distribution for the OAM mode *l*=3, respectively, and the third and fourth columns indicate simulated Ex field intensity and phase distribution for the OAM mode *l*=4, respectively. The first and second rows indicate the vortex beam generated from the metasurface with and without meta-lens structure, respectively.


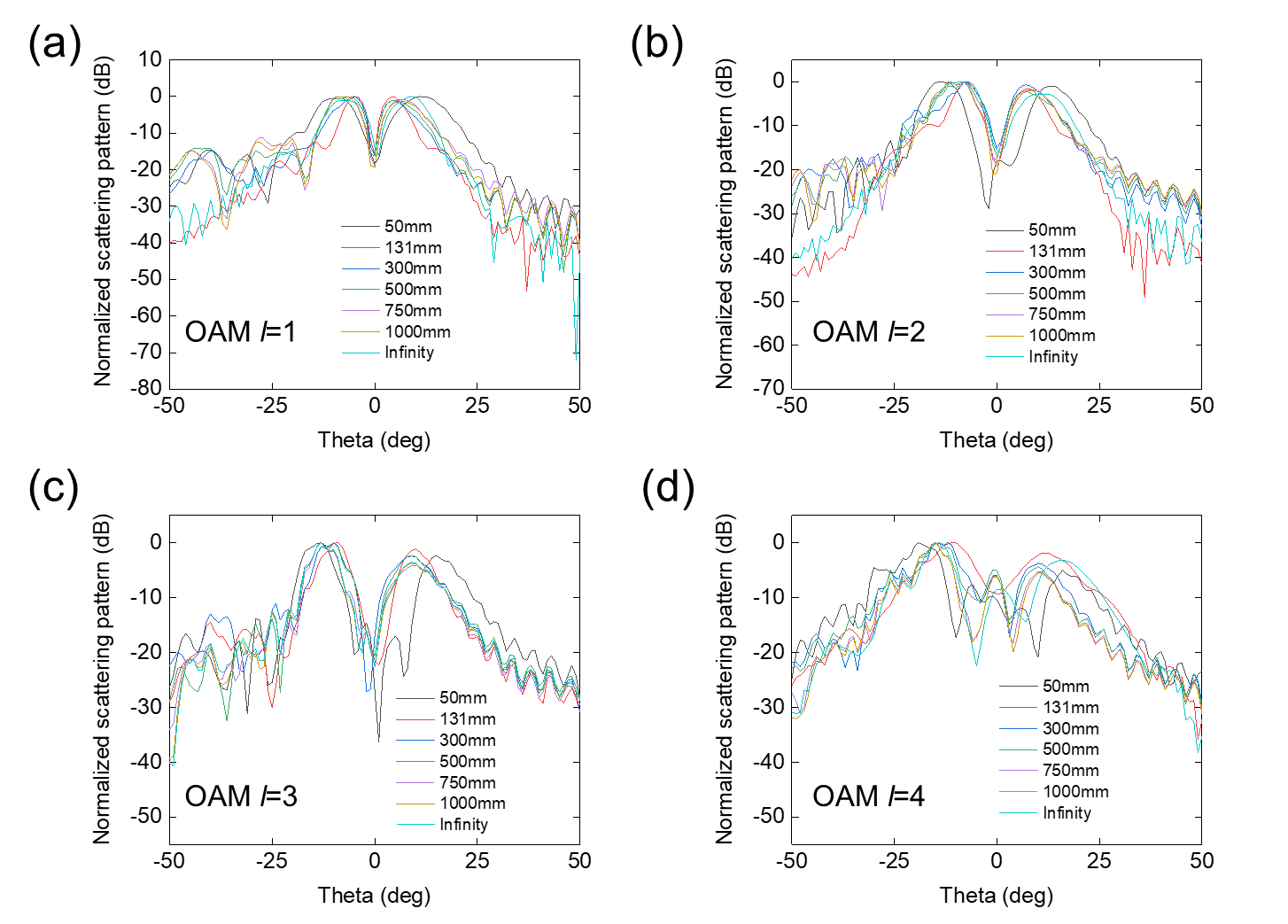


**Figure S11**. Simulation results of the normalized 2D scattering patterns for the OAM mode *l*=1(a), 2 (b), 3 (c), 4 (d) from the metasurface with meta-lens pattern with seven different focal lengths (F=50 mm, 131 mm, 300 mm, 500 mm, 750 mm, 1000 mm, and infinity).

**Figure S12**. Simulation results of the divergence angle of the OAM mode l=1, 2, 3, 4 from the metasurface with meta-lens pattern with seven different focal lengths (F=50 mm, 131 mm, 300 mm, 500 mm, 750 mm, 1000 mm, and infinity).
